# Molecular Detection of Hemoparasites in Hematophagous Insects Collected from Livestock Farms in Northeastern Thailand

**DOI:** 10.3390/insects16020207

**Published:** 2025-02-14

**Authors:** Pairpailin Jhaiaun, Apiraya Rudeekiatthamrong, Wissanuwat Chimnoi, Giang Thi Nguyen, Ruttayaporn Ngasaman, Jumnongjit Phasuk, Ketsarin Kamyingkird

**Affiliations:** 1 Department of Parasitology, Faculty of Veterinary Medicine, Kasetsart University, Lad Yao, Chatuchak, Bangkok 10900, Thailand; pairpailin.j@gmail.com (P.J.); wissanuwat.c@ku.th (W.C.); giangthi.n@ku.th (G.T.N.); 2 Faculty of Veterinary Science, Prince of Songkla University, Songkhla 90110, Thailand; ruttayaporn.n@psu.ac.th

**Keywords:** anaplasmosis, diseases transmission, hematophagous insects, molecular assay, theileriosis, trypanosomiasis, vector-borne parasitic diseases (VBPDs), *Stomoxys*, *Tabanus*

## Abstract

Vector-borne parasitic diseases (VBPDs) are diseases that have a major impact on the health and productivity of livestock. VBPDs, including trypanosomosis, theileriosis, and anaplasmosis, are caused by blood parasites and Rickettsiales infections that can be transmitted by biting insects. The control and elimination of VBPDs is difficult and challenging. This study contributed to better understanding the impact of hematophagous insects on carrying pathogens to livestock by investigating the detection of *Trypanosoma evansi*, *Theileria* spp. and *Anaplasma* spp. in hematophagous insects collected from five livestock farms in Northeastern Thailand using polymerase chain reaction (PCR). The *Stomoxys* and *Tabanus* samples were collected using Nzi and Vavoua traps. Morphological and molecular identifications of hematophagous insects were performed. The *Stomoxys* samples were dissected into head and abdomen parts, while the *Tabanus* samples were dissected into head, salivary glands, and abdomen parts before DNA extraction individually. The host blood meal in the DNA of each part of the hematophagous insects was identified using PCR targeting the prepronociceptin (*PNOC*) gene. PCR detection of *T. evansi*, *Theileria* spp., and *Anaplasma* spp. were performed by targeting the *ITS2*, *18s rRNA,* and *msp4* genes, respectively. In total, 131 insect samples were collected, with *Stomoxys calcitrans* being the most common species at 40 (30.53%), followed by *Tabanus* spp. at 14 (10.69%) and the other *Stomoxys* spp. at 7 (5.34%), respectively. The hemoparasite detection results identified *Theileria* spp. in the abdomen (21.43%; 3/14) of three *Tabanus* samples. Neither *Anaplasma* spp. nor *T. evansi* were detected in this study. Additionally, host blood meal DNA was found in the abdomen of *Stomoxys calcitrans* (4.17%; 1/24) and *Stomoxys* spp. (20%; 1/5). For the *Tabanus* spp., host blood meal DNA was detected in the head (7.14%; 1/14), salivary glands (7.14%; 1/14), and abdomen (14.29%; 2/14). These results highlighted that *Tabanus* spp. might play an important role in *Theileria* transmission.

## Simple Summary

Vector-borne parasitic diseases (VBPDs) have a major impact on livestock health and productivity and are transmitted by biting insects such as *Stomoxys* and *Tabanus*. This study used PCR techniques to detect *Trypanosoma evansi*, *Theileria* spp., and *Anaplasma* spp. in insects from livestock farms in Northeastern Thailand. While *Theileria* spp. were found in the abdomen of *Tabanus* spp., neither *Anaplasma* spp. nor *T. evansi* were detected. These findings suggested that *Tabanus* spp. play a key role in *Theileria* transmission among livestock.

## 1. Introduction

Hematophagous insect infestation in livestock, including various flies, fleas, lice, ticks, and mites, are major vectors for transmitting bacterial, viral, Rickettsiales, and protozoan pathogens and cause several important diseases in animals [1]. Vector-borne parasitic diseases (VBPDs) include, but are not limited to, anopheles-borne diseases (malaria); tick-borne diseases (babesiosis, piroplasmosis); snail-borne diseases (schistosomiasis); sand fly-borne diseases (leishmaniasis); tsetse fly-borne diseases (African trypanosomiasis); and triatomine bug-borne diseases (chagas disease) [2]. In animals, VBPDs have a high impact on animal health and production. Most of the VBPDs in animals are blood parasitic diseases that can be carried by ticks (tick-borne diseases) and biting flies (fly-borne diseases). Important VBPDs in animals, such as trypanosomosis, theileriosis, and anaplasmosis, are difficult to control and eliminate, and so they are of veterinary importance in Thailand.

Animal trypanosomosis or surra is caused by *Trypanosoma* (*T*.) *evansi* (Steel, 1885), which is one of the most prominent VBPDs of cattle and equids in Thailand [3]. *T. evansi* is mainly mechanically transmitted by biting flies, including *Stomoxys* (Geoffroy, 1762), *Tabanus* (Linnaeus, 1758), and *Haematopota* (Meigen, 1803) [3,4]. One study reported that *T. evansi* could be mechanically transmitted by *Tabanus* briefly when the insect feeds on an infected animal and then immediately feeds on another animal [5]. In addition, the transmission of *T. evansi* is relevant to parasitaemia, the number of biting insects, insect size, morphology, and the density of insect infestation [6]. Furthermore, *T. evansi* may lead to abortion, which is a major concern in the dairy cattle production industry [7]. Molecular techniques are useful tools that allow for the precise detection of *Trypanosoma* species by amplifying specific DNA sequences, making such techniques a highly sensitive and accurate method for detection of *T. evansi* infection [8,9].

Theileriosis is a disease caused by apicomplexan protozoan parasites in the genus *Theileria* and is predominantly biologically transmitted by ticks [10,11]. Theileriosis has a major impact on animal health and livestock production, and it can cause severe physical and immunological damage to cattle [12,13,14]. In Thailand, *Theileria* was first reported in cattle in 1971 [15]. Bovine theileriosis in Thailand is mostly caused by benign groups that include *Theileria sergenti* (Yakimoff and Dekhtereff, 1930), *Theileria buffeli* (Schein, 1908), and *Theileria orientalis* (Yakimoff and Soudatschenkoff, 1931) [16,17,18]. Clinical symptoms of cattle infected with the *Theileria* benign group can be mild or absent [19], making diagnosis difficult. The 18s ribosomal RNA (*18s rRNA*) gene has served as an effective genetic marker for identifying and characterizing blood parasites, enabling reliable detection and genetic analysis of parasite DNA in horses [20], lions [21], and biting flies [22]. It has long been known that *Theileria* spp. is predominantly biologically transmitted by ticks [23]. However, molecular detection of *Theileria* DNA in *Stomoxys* has also been reported [22,24,25,26,27].

Anaplasmosis is caused by alpha-proteobacteria pathogens of the order Rickettsia. Anaplasmosis is one of the most prominent tick-borne diseases (TBDs) in animals that affects animal health and livestock production [28]. *Anaplasma* infections in cattle can be characterized by clinical symptoms such as haemolytic anemia, high fever, weight loss, abortion, decreased milk production, and death [29]. Ruminants have been reported to be infected by several species in the genus *Anaplasma*, such as *Anaplasma marginale* (Theiler, 1910), *Anaplasma centrale* (Theiler, 1911), *Anaplasma bovis* (Donatien, 1963), *Anaplasma platys* (Harvey, 1978), *Anaplasma ovis* (Laveran and Nocard, 1912), and *Anaplasma phagocytophilum* (Dumler, 2001) [30,31]. Anaplasmosis is biologically transmitted by ticks [29,32,33]. However, mechanical transmission can occur via blood-sucking insects, resulting in contamination of blood [34]. Notably, several *Tabanus* species have been identified as mechanical vectors, and these species may potentially transmit *A. marginale* among cattle [35]. Molecular detection of *Anaplasma* spp. has widely been used in anemic cattle, based on targeting the major surface proteins (*msp*) genes such as *msp4* [30,31,36]. In addition, the *msp4* can be used for the detection of *Anaplasma* spp. in hematophagous insects.

PCR has become the preferred species-specific molecular diagnostic assay in veterinary parasitology for detecting pathogens with high sensitivity [19,37,38]. It is more sensitive than the thin blood smear technique for confirming theileriosis [39,40], trypanosomosis [41,42] and anaplasmosis [30,43]. PCR can be used for the detection of pathogens in the infected host as well as insect vectors [44]. VBPD outbreaks have been reported on Thai small-scale livestock farms several times. The aim of the current study was to better understand the role of hematophagous insects in VBPD transmission in Thailand by detecting *T. evansi*, *Theileria* spp., and *Anaplasma* spp. using a molecular assay on samples from hematophagous insects collected on livestock farms in Northeastern Thailand.

## 2. Materials and Methods

### 2.1. Study Design and Data Collection

**Figure 1:**
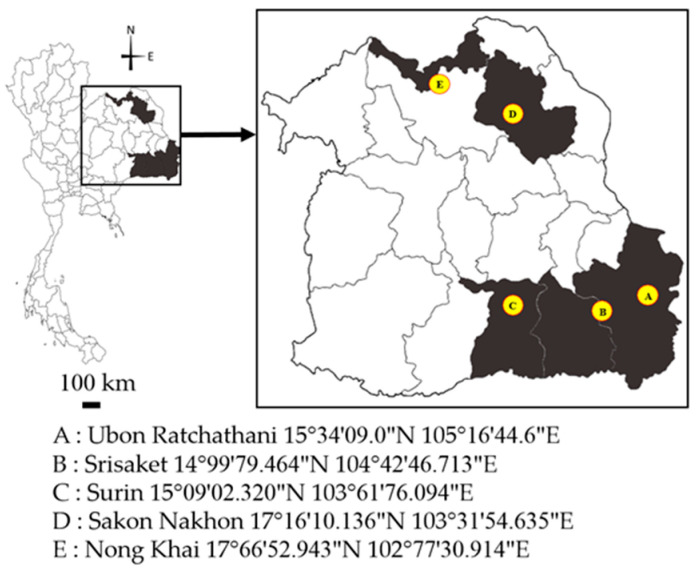
Study area and insect trapping sites.

This study was designed to collect hematophagous insects from small-scale livestock farms in five provinces of Northeastern Thailand. The insect trapping sites included Muang District, Ubon Ratchathani (15°34′09.0″ N 105°16′44.6″ E); Muang District, Srisaket province (14°99′79.464″ N 104°42′46.713″ E); Chom Phra District, Surin province (15°09′02.320″ N 103°61′76.094″ E); Waritchaphum District, Sakon Nakhon province (17°16′10.136″ N 103°31′54.635″ E), and Sakhrai District, Nong Khai province (17°66′52.943″ N 102°77′30.914″ E) (Figure 1). One small-scale livestock farm per province was selected and the latitude and longitude details were recorded for each farm.

### 2.2. Hematophagous Insect Sample Collection

**Figure 2:**
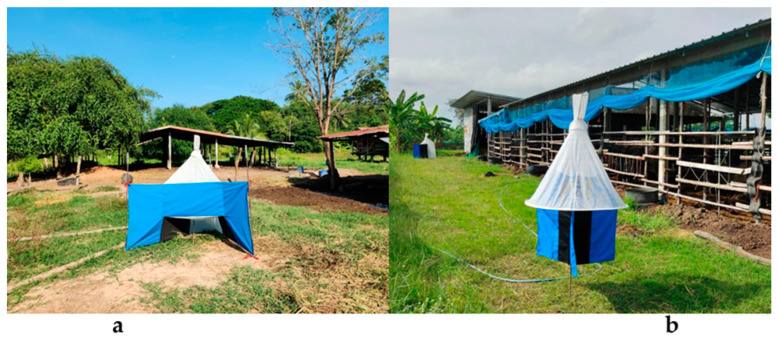
Nzi trap (**a**) and Vavoua trap (**b**).

*Stomoxys* and *Tabanus* samples were collected using Nzi and Vavoua traps (Figure 2), as described elsewhere [45,46]. The traps were randomly placed near animal stables; the distance of each trap was at least 5 m apart from each other and the traps were placed at 5–10 cm above the ground during the day for 6 h (9.00 a.m. to 14.00 p.m.) [46]. The *Stomoxys* and *Tabanus* collected from these traps were preserved in sterile tubes with 80% ethanol [46] and then transferred to the Department of Parasitology, Faculty of Veterinary Medicine, Kasetsart University, Bangkok, Thailand, for further identification.

### 2.3. Morphological Identification, Dissection, and DNA Extraction

Morphological identification of *Stomoxys* and *Tabanus* samples was based on an identification key, as described elsewhere [47,48,49].

**Figure 3:**
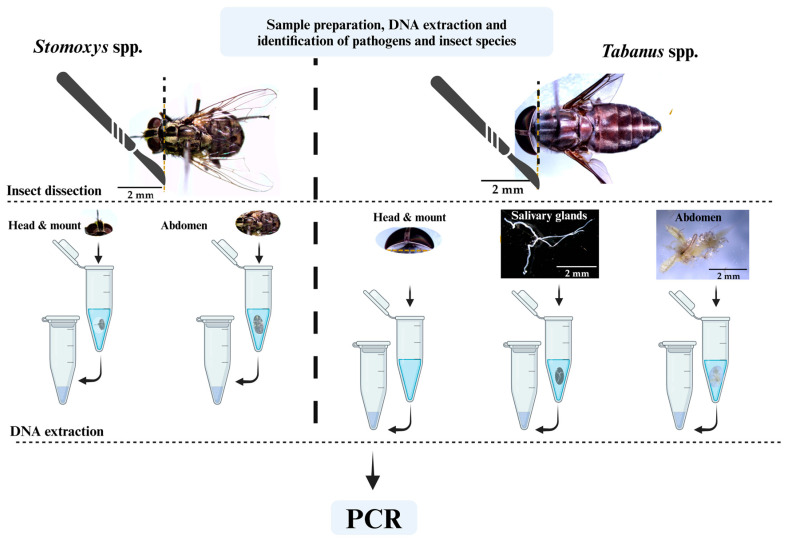
A flow chart showing sample preparation, insect dissection, and DNA extraction.

The *Stomoxys* and *Tabanus* samples were individually immersed in sterile distilled water to remove excess ethanol before dissection. The *Stomoxys* samples were carefully dissected into 2 parts, while the *Tabanus* samples were dissected into 3 parts (Figure 3). The dissected parts were individually used for DNA extraction with a tissue DNA extraction kit (Monarch^®^; Ipswich, MA, USA), as recommended by the manufacturer. Subsequently, genomic DNA concentration and quality values were measured using an Eppendorf BioSpectrophotometer (Eppendorf AG, Hamburg, Germany) at 260 and 280 nanometers and stored at −20 °C until further analysis. DNA extraction and PCR procedures were carefully conducted by the first author, an entomologist who was well trained and experienced. No automated machine was used in this study.

### 2.4. Molecular Detections of Hemoparasites

#### 2.4.1. PCR Detection of *T. evansi*

PCR detection of *T. evansi* was performed using the *ITS2* gene ITS2F (5′-TGT CAC GCA TAT ACG TGT GTG-3′) and ITS2R (5′-TAC ACA CAT ACA CAC TAT CCG-3′), as described elsewhere [50]. The PCR master mix was prepared in totals of 10 μL per reaction, containing 5 μL of *Taq 2X* master mix buffer (New England Biolabs; Ipswich, MA, USA), 0.2 μL of each primer (20 μM), 3.6 μL of distilled water (DW), and 1 μL of DNA template. The thermocycler conditions were initial denaturation at 95 °C for 2 min, 30 cycles of 95 °C for 30 s, 52.8 °C for 30 s, 72 °C for 30 s, and a final extension at 72 °C for 2 min. The *T. evansi* positive control was derived from cattle blood and distilled water was used as a negative control.

#### 2.4.2. PCR Detection of *Theileria* spp.

The *Theileria 18s rRNA* gene was used to amplify an approximately 500 bp fragment based on the primers BJ1 (forward: 5′-GTCTTGTAATTGGAATGATGG-3′) and BN2 (reverse: 5′-TAGTTTATGGTTAGGACTACG-3′) [27]. The PCR master mix was prepared in a total of 10 μL containing 5 μL of *Taq 2X* master mix buffer (New England Biolabs; Ipswich, MA, USA), 0.2 μL of each primer (20 μM), 3.6 μL of DW, and 1 μL of DNA template. The thermocycler conditions were initial denaturation at 94 °C for 10 min, 35 cycles of denaturation at 94 °C for 1 min, annealing at 55 °C for 1 min, elongation at 72 °C for 2 min, and a final extension at 72 °C for 5 min. The *Theileria orientalis* positive control was derived from cattle blood and distilled water was used as a negative control.

#### 2.4.3. PCR Detection of *Anaplasma* spp.

The *Anaplasma* spp. *msp4* gene were amplified using 0.2 μL of each primer (20 μM) (MSP45: 5′ GGG AGC TCC TAT GAA TTA CAG AGA ATT GTT TAG GGA GCT CCT ATG AAT TAC AGA GAA TTG TTT AC 3′ and MSP43: 5′ CCG GAT CCT TAG CTG AAC AGG AAT CTT GC 3′). The PCR master mix was prepared in a total of 10 μL per reaction containing 5 μL of *Taq 2X* master mix buffer (New England Biolabs, Ipswich, MA, USA), 3.6 μL of DW, and 1 μL of DNA template. The thermocycler conditions were initial denaturation at 95 °C for 2 min, 40 cycles of 95 °C for 3 s, 60 °C for 3 s, 72 °C for 1 min, and a final extension at 72 °C for 3 min, as described elsewhere [51]. The *Anaplasma marginale* positive control was derived from cattle blood and distilled water was used as a negative control.

### 2.5. Molecular Detections of Hemoparasites

#### Blood Meal Identification

The presence and identification of host blood meal in each part of the sampled hematophagous insects (*Stomoxys* and *Tabanus*) was conducted using PCR targeting the *PNOC* genes, as described elsewhere [52]. The primer set of PNOC-Forward primer (forward: 5′-GCA TCC TTG AGT GTG AAG AGA A-3′) and PNOC-Reverse primer (reverse: 5′-TGC CTC ATA AAC TCA CTG AAC C-3′) was used to amplify 330 bp. The PCR mixture was prepared in a total of 10 µL per reaction containing 5 μL of *Taq 2X* master mix buffer (New England Biolabs, Ipswich, MA, USA), 0.2 μL of each primer (10 μM), 3.6 μL of DW, and 1 μL of DNA template. The thermocycler conditions were initial denaturation at 95 °C 5 min, 35 cycles of 95 °C for 30 s, 55 °C for 30 s, 72 °C for 45 s, and a final elongation at 72 °C for 5 min, as described elsewhere [52]. The positive control was derived from cattle blood and distilled water was used as a negative control.

### 2.6. Sequencing and Bioinformatics Analysis

The PCR products were purified using a gel extraction kit (Mecherey-Nagel, Dueren, Germany) and subsequently used for nucleotide sequencing. Nucleotide sequence quality was monitored and trimmed using the Unipro UGENE software (version 41.0) [53]. The obtained nucleotide sequences were compared to reference sequences using the Basic Local Alignment Search Tool (BLAST) in the National Center for Biotechnology Information (NCBI) (https://blast.ncbi.nlm.nih.gov, accessed on 26 October 2024). A phylogenetic tree was constructed using a maximum likelihood method in the Mega software (version 11).

## 3. Results

### 3.1. Hematophagous Insects Collected

In total, 131 hematophagous insect samples were collected from five selected small-scale livestock farms. Based on the morphological identification, there were 40 samples of *Stomoxys calcitrans* (30.53%), 14 samples of *Tabanus* spp. (10.69%), and 7 samples of *Stomoxys* spp. (5.34%), as shown in Table 1. In addition, there were house flies (*Musca domestica*) and midges (*Culicoides* spp.) collected from the Nzi and Vavua traps.

### 3.2. Molecular Detection of Hemoparasites in Hematophagous Insects

*Theileria* spp. were detected in the abdomen (21.43%; 3/14). A sequencing analysis confirmed that the species of *Theileria* found in the abdomen part were *T. orientalis* (98.96% identity, accession no. PQ539410) and *Theileria sinensis* (Bai, 2019) (96.20% identity, accession no. PQ539409). No *Anaplasma* spp. or *T. evansi* were detected in either the *Stomoxys* or *Tabanus* samples collected in this study (Table 2). Furthermore, there were no hemoparasites detected in any parts of the *Stomoxys* collected in this study.

### 3.3. Presence of Host Blood in Body Parts of Hematophagous Insects and Bioinformatic Analysis

Host blood meal DNA was detected in the head, salivary gland, and abdomen DNA samples selected from six *Stomoxys* and *Tabanus* insect samples. In the abdomen parts of *Stomoxys calcitrans* and *Stomoxys* spp., there was host blood meal DNA detected in 4.17%; 1/24 and 20%; 1/5 samples, respectively.

For the *Tabanus* spp., host blood meal DNA was detected in the head (1/14; 7.14%), salivary gland (1/14; 7.14%), and abdomen (2/14; 14.29%), as shown in Table 3. Based on the results from the sequencing for detection of host blood meal DNA in the hematophagous insects, there were *Homo sapiens* (Linnaeus, 1758) (99.66% identity) and *Bos indicus* (Linnaeus, 1758) (99.66% and 99.67% identity).

*Theileria* were detected in the abdomen part of *Tabanus* (Table 3). The confirmation of *Theileria* species revealed that one *Tabanus* abdomen sample carried *T. orientalis* with 98.96% identity (accession no. PQ539410) and the other *Tabanus* abdomen sample carried *T. sinensis* with 96.20% identity (accession no. PQ539409). However, there were no *Theileria* species detected in the salivary gland of the *Tabanus* samples (Table 3). Notably, one positive identification of *Theileria* in a *Tabanus* abdomen part that carried *Theileria* sp. with 91.28% identity (accession no. not obtained) also contained host blood (Table 3). The *Theileria* sp. detected in the *Tabanus* abdomen sample might have resulted from host blood meal.

A comparison of individual hematophagous samples that were positive for *Theileria* and host blood meal detection is shown in Table 4. The result showed that one *Tabanus* abdomen (T3) was positive for both *Theileria* (sequence was not able to specify *Theileria* species) and host blood meal (*Bos indicus*) (Table 4). This result might reveal that the *Theileria* positive in *Tabanus* (T3) might come from the host blood meal. However, there were three *Tabanus* (T2, T11 and T1) that were positive with *Theileria* spp. but not positive for host blood meal (Table 4). These three *Tabanus* samples confirmed no host blood meal contamination and also indicated a *Theileria* presence in the *Tabanus* abdomen part.

The presence of host blood meal in the head, salivary glands, and abdomen parts of hematophagous insects helps to confirm the source of pathogen detection. The presence of blood meal in the head of *Tabanus* (T14) reveals the possibility that a pathogen from the blood contained in the mouth part can be mechanically transmitted. The presence of host blood meal in the abdomen of *Tabanus* (T3, T7) and *Stomoxys* (S8, S18) indicate the role of *Tabanus* and *Stomoxys* as blood-sucking insect vectors in livestock farming. However, the presence of host blood in the salivary gland of *Tabanus* (T10) is unusual. Careful dissection of hematophagous insects is recommended for future studies.

### 3.4. Phylogenetic Analysis

**Figure 4:**
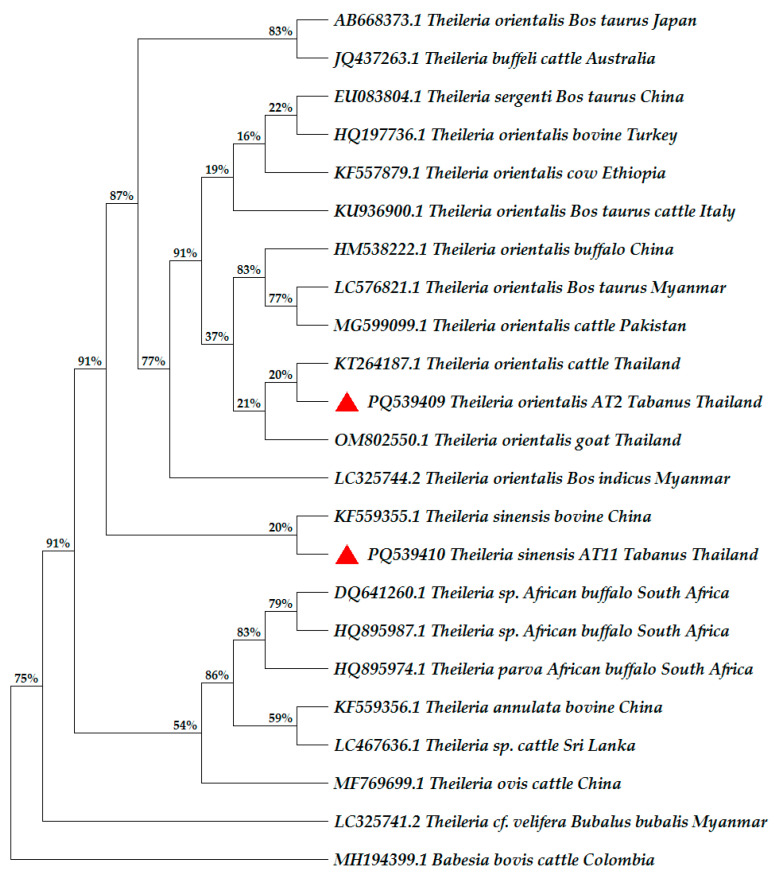
A phylogenetic tree based on *18s rRNA* gene sequences of *T. orientalis* and *T. sinensis* detected in a *Tabanus* abdomen in this study compared to reference sequences from GenBank by using the maximum likelihood method at 1000 bootstraps. *Babesia bovis* was used as the outgroup. The sequences generated in this study are represented by a red symbol.

A phylogenetic analysis of *T. orientalis* detected in a *Tabanus* abdomen from this study (accession no. PQ539410) showed that they were similar to the reference sequences of *T. orientalis* isolated from cattle in Thailand, Myanmar, and Pakistan (Figure 4), while *T. sinensis* sequences obtained from a *Tabanus* abdomen in this study (accession no. PQ539409) confirmed that they were similar to the reference sequence of *T. sinensis* from bovine in China (Figure 4). The outgroup was *Babesia bovis* (Babes, 1888) from cattle in Colombia (accession no. MH194399.1).

## 4. Discussion

The *Stomoxys* genus is classified in the order Diptera and the family Muscidae [54], with the members of the Muscidae being divided into 200 genera and 5000 species [55]. In Thailand, 11 species of *Stomoxys* in 5 genera have been reported [56]. Those 5 genera (*Stomoxys*, *Haematobosca* (Bezzi, 1907), *Haematobia* (Lepeletier and Serville, 1828), *Haematostoma* (Malloch, 1932), and *Stygeromyia* (Austen, 1907)) are major vectors in the livestock industry [57]. In Thailand, six species have been reported of *Stomoxys* flies (*S. calcitrans* (Linnaeus, 1758), *Stomoxys indicus* (Picard, 1908), *Stomoxys uruma* (Shinonaga and Kano, 1966), *Stomoxys pulla* (Austen, 1909), *Stomoxys sitiens* (Rondani, 1873), and *Stomoxys bengalensis* (Picard, 1908) [49,58,59,60,61]. The hematophagous insect samples in the current study were collected in November, which is the dry season. However, another study reported patterns of greater abundance of *Stomoxys* flies during the rainy season (May–June) [61]. On the other hand, there were reported pathogens detected in *Stomoxys* flies [22] and *Tabanus* flies. Previous studies have also mentioned that biting flies may also act as potential vectors [62].

*Tabanus* spp. or horse flies comprise more than 100 genera and 4000 species worldwide [63]. Among those 4000 species, there are at least 80 *Tabanus* species that have been reported in Thailand [47]. Horse flies (*Tabanus* spp.) have been reported in Thailand, with the most abundant species being *Tabanus striatus* (Fabricius, 1787), *Tabanus megalops* (Walker, 1854), *Tabanus rubidus* (Wiedemann, 1821), *Tabanus tamthaiorum* (Burton, 1978), and *Tabanus oxybeles* (Burton, 1978) [64]. The current study identified *T. orientalis* and *T. sinensis* in the *Tabanus* spp. samples, which was consistent with a previous study that reported that *Theileria* was the most prevalent parasite species found in beef cattle in Northern and Northeastern Thailand [65]; therefore, *T. sinensis* was also circulating within the cattle population in Thailand [66]. However, *Anaplasma* spp. and *Trypanosoma* spp. were not detected in the biting insect samples collected in the current study, which might have been due to the low prevalence of the corresponding diseases in livestock in the area as well as the low number of biting insect samples collected in this study. A larger sample size may increase the detection of parasites in biting insects. In contrast, previous studies reported *A. marginale* in *Tabanus* spp. using whole-body DNA extraction [67], whereas our study involved dissecting *Tabanus* into three separate parts for analysis individually.

The blood meal of hematophagous insects is crucial for the maturation of eggs and the deposition of yolk in developing oocytes [68]; notably, larger female insects consume more blood during feeding, which enhances their egg production capacity [69]. Identification of blood meal and pathogens in hematophagous insects helps to clarify the true vector roles by distinguishing between active infections and the presence of blood meal [70]. In this study, we used a *PNOC* gene to confirm the presence of host blood meal in the insect DNA samples that were consumed within 48 h [52]. Based on the results from the current study, there were two abdominal parts of *Tabanus* samples (T3 and T7) containing host blood meal in which one *Tabanus* (T3) sample was also detected *Theileria* sp. This indicated that the *Theileria* found in the *Tabanus* sample might have come from the *Theileria*-infected blood meal consumed by the insect. The findings from this study suggest that the detection of pathogens in hematophagous insects should also help identify the host blood meal contamination in future studies. The presence of host blood in the head of *Tabanus* in this study indicates that pathogen transmission from a blood-contaminated *Tabanus* mouthpart could carry and transmit pathogens via the biting behavior of *Tabanus*. This finding relates to other studies that reported that the mechanical transmission of pathogens from one to another host via localized irritation was caused by *Tabanus* mouthpart penetration [71,72]. However, we have also detected host blood DNA in the salivary gland of *Tabanus* (T10) in our study. In general, blood meal should not draw from the mouth part to the salivary gland. Even the insect dissection was carefully performed using a sterile technique; however, there might be a possibility of blood contamination from the gut or during the dissection process. Therefore, careful dissection and the use of of disposable and sterile dissection equipment for the dissection of fully fed hematophagous insects is required in further studies.

In general, *Theileria* is biologically transmitted by ticks. However, previous studies have highlighted high prevalences of *Theileria* infection in Thailand. Identification of vector-borne parasites, including *Theileria*, in biting insects such as *Stomoxys* and *Tabanus*, which are highly distributed hematophagous insects in Thailand, might increase our understanding of *Theileria* transmission routes. Detection of *Theileria* in the hematophagous insect parts in this study reveal that the *Theileria 18s rRNA* gene sequences in this study were confirmed as *T. oriantalis* and *T. sinensis*. Previously, *Theileria orientalis* types 1–8 and N1–N3 [73] have been reported based on the major piroplasm surface protein (*MPSP*) gene. In Thailand, *T. orientalis* in Thai cattle *were* reported as type 1, 3, 5, 6, 7, and N3 [72]. However, the type of *T. orientalis* in *Tabanus* in this study has not been identified. Using the *MPSP* gene for detection of the *Theileria* type in hematophagous insects might be applied in further studies. In Thailand, *T. orientalis* was also the most prevalent parasite in domestic ruminants and bullfighting cattle [18,65]. For instance, research conducted in Northeastern Thailand has previously confirmed the presence of *T. sinensis* and *T. orientalis* [74], which is related to the findings of this study. However, the limitation of this study was its small sample size due to the short sampling duration and the limited number of insect traps. More sampling sites and a larger number of farms and insect samples should be utilized in future studies. Hence, cytochrome b gene targeting host blood meal can be applied in further studies to detect digested blood meal consumed after more than 48 h in the abdomen of hematophagous insects.

## 5. Conclusions

This study investigated the possibility that *Tabanus* may play an important role in the transmission of *Theileria* and other pathogens. The biting insects control measures should be promoted to reduce vector-borne parasitic disease transmission in livestock farming.

## Figures and Tables

**Table 1:** Numbers of hematophagous insects collected.

Province	Srisaket	Ubon Ratchathani	Nong Khai	Surin	Sakon Nakhon	Total
*Stomoxys calcitrans*	4	15	15	0	6	40
*Stomoxys* spp.	1	1	0	4	1	7
*Tabanus* spp.	0	2	9	1	2	14
Total	5	18	24	5	9	61

**Table 2:** Molecular detection of hemoparasites in the hematophagous insect samples collected from livestock farms.

Molecular Detection of Hemoparasites	*Stomoxys calcitrans* (n = 24)		*Stomoxys* spp. (n = 5)		*Tabanus* spp. (n = 14)			Total (n = 43)
	Head	Abdomen	Head	Abdomen	Head	Salivary Gland	Abdomen	
*T*. *evansi*	0	0	0	0	0	0	0	0
*Theileria* spp.	0	0	0	0	0	0	3 (21.43%)	3 (6.97%)
*Anaplasma* spp.	0	0	0	0	0	0	0	0

**Table 3:** The presence of host blood DNA in hematophagous insects and the confirmation of hemoparasite species using sequencing analysis.

Type of Hematophagous Insects	Body Part of Hematophagous Insects	Presence of Blood Meal (%)	Presence of *Theileria* spp. (P/Total)	Pathogen Species (n)	Identity (%)	Accession no.
*Stomoxys cacitrans*	Head	0/24 (0.00%)	0/24	-	-	-
*Stomoxys* spp.	Head	0/5 (0.00%)	0/5	-	-	-
*Tabanus* spp.	Head	1/14 (7.14%)	0/14	-	-	-
*Stomoxys calcitrans*	Abdomen	1/24 (4.17%)	0/24	-	-	-
*Stomoxys* spp.	Abdomen	1/5 (20%)	0/5	-	-	-
*Tabanus* spp.	Salivary	1/14 (7.14%)	0/14	-	-	-
*Tabanus* spp.	Abdomen	2/14 (14.29%)	3/14	*Theileria orientalis* (1) *Theileria sinensis* (1) *Theileria* sp. (1) *	98.96% 96.20% 91.28%	PQ539410 PQ539409 NA

NA = not available, P = positive, n = number of sample; * = blood meal.

**Table 4:** Comparison of *Theileria* positive and blood meal positive in each of the hematophagous insects.

Individual Insects Samples (ID) *Theileria* spp.	*Theileria* spp.			Host Blood Meal		
	Head	Salivary Gland	Abdomen	Head	Salivary Gland	Abdomen
*Tabanus* (T2)	−	−	+ ^a^	−	−	−
*Tabanus* (T3)	−	−	+ ^b^	−	−	+ ^d^
*Tabanus* (T11)	−	−	+ ^c^	−	−	−
*Tabanus* (T7)	−	−	−	−	−	+
*Tabanus* (T10)	−	−	−	−	+	−
*Tabanus* (T14)	−	−	−	+	−	−
*Stomoxys* spp. (S8)	−	−	−	−	ND	+ ^e^
*Stomoxys calcitrans* (S18)	−	−	−	−	ND	+ ^f^

+ = Positive, − = Negative, ND = not detected; sequencing results; ^a^ *Theileria orientalis* (accession no. PQ539410), ^b^ *Theileria* sp. (accession no. not available), ^c^ *Theileria sinensis* (accession no. PQ539409), ^d^ *Bos indicus* (99.66% identity), ^e^ *Homo sapiens* (99.66% identity), ^f^ *Bos indicus* (99.67% identity).

## Data Availability

All relevant data are included in the article.
